# A systematic approach identifies FOXA1 as a key factor in the loss of epithelial traits during the epithelial-to-mesenchymal transition in lung cancer

**DOI:** 10.1186/1471-2164-14-680

**Published:** 2013-10-04

**Authors:** Haiyun Wang, Clifford A Meyer, Teng Fei, Gang Wang, Fan Zhang, X Shirley Liu

**Affiliations:** 1 School of Life Science and Technology, Tongji University, Shanghai 200092, China; 2 Department of Biostatistics and Computational Biology, Dana-Farber Cancer Institute and Harvard School of Public Health, Boston, MA 02215, USA; 3 Tongji University Advanced Institute of Translational Medicine, Tongji University, Shanghai 200092, China; 4 Department of Medical Oncology, Division of Molecular and Cellular Oncology, Dana-Farber Cancer Institute and Harvard Medical School, Boston, MA 02215, USA; 5 Clinical Translational Research Center, Shanghai Pulmonary Hospital, School of Life Science and Technology, Tongji University, Shanghai 200092, China

**Keywords:** The epithelial-to-mesenchymal transition, Lung cancer, ChIP-Seq, FOXA1

## Abstract

**Background:**

The epithelial-to-mesenchymal transition is an important mechanism in cancer metastasis. Although transcription factors including SNAIL, SLUG, and TWIST1 regulate the epithelial-to-mesenchymal transition, other unknown transcription factors could also be involved. Identification of the full complement of transcription factors is essential for a more complete understanding of gene regulation in this process. Chromatin immunoprecipitation-sequencing (ChIP-Seq) technologies have been used to detect genome-wide binding of transcription factors; here, we developed a systematic approach to integrate existing ChIP-Seq and transcriptome data. We scanned multiple transcription factors to investigate their functional impact on the epithelial-to-mesenchymal transition in the human A549 lung adenocarcinoma cell line.

**Results:**

Among the transcription factors tested, impact scores identified the forkhead box protein A1 (FOXA1) as the most significant transcription factor in the epithelial-to-mesenchymal transition. FOXA1 physically associates with the promoters of its predicted target genes. Several critical epithelial-to-mesenchymal transition effectors involved in cellular adhesion and cellular communication were identified in the regulatory network of FOXA1, including FOXA2, FGA, FGB, FGG, and FGL1. The implication of FOXA1 in the epithelial-to-mesenchymal transition via its regulatory network indicates that FOXA1 may play an important role in the initiation of lung cancer metastasis.

**Conclusions:**

We identified FOXA1 as a potentially important transcription factor and negative regulator in the initial stages of lung cancer metastasis. FOXA1 may modulate the epithelial-to-mesenchymal transition via its transcriptional regulatory network. Further, this study demonstrates how ChIP-Seq and expression data could be integrated to delineate the impact of transcription factors on a specific biological process.

## Background

The epithelial-to-mesenchymal transition (EMT) is an important mechanism for cancer metastasis [1], the cause of 90% of deaths from solid tumors [2]. During EMT, epithelial cells lose epithelial characteristics and gain a mesenchymal morphological phenotype with the accompanying migration and invasion characteristics [3]. Since invasion of tumor cells into the bloodstream is the first step of metastasis, EMT enables the tumor cells to migrate and invade [1].

EMT is induced by expression of transcription factors (TFs), such as SNAIL [4,5], SLUG [5], ZEB1 [6,7], ZEB2 [8], E47 [5,9], TWIST1 [10], FOXC2 [11] and Goosecoid [12]. These TFs suppress critical epithelial cell traits, permitting the transformation to mesenchymal cells to occur. In cancer, overexpression of TFs promotes EMT in tumor cells. Although most tumors seem to undergo EMT because of overexpression of 3 particular families of TFs (SNAIL, ZEB, and TWIST) [13], additional EMT-regulating TFs may remain to be identified.

Because TFs are essential for the regulation of gene expression through their interactions with regulatory DNA sequences, sites where they bind to DNA can be detected by chromatin immunoprecipitation coupled with sequencing (ChIP-Seq). Indeed, ChIP-Seq technique can be used to infer gene regulatory mechanism in specific biological processes [14]. Further, the increasing amount of TF and DNA binding information in public databases can help inform the role of TFs in cancer and in EMT in particular.

We systematically analyzed a large collection of ChIP-Seq TF binding profiles in the A549 lung adenocarcinoma cell line. We evaluated the functional impact of TFs during EMT by integrating ChIP-Seq data with gene expression data in A549 cells treated with TGF-beta to induce EMT. We then assessed the functional impact of these expression changes on specific biological processes. This approach identified the forkhead box protein A1 (FOXA1) as the most significant TF during EMT in A549 lung cancer cells. Functional analysis suggests that FOXA1 is involved in the loss of cell adhesion and cell communication during the initiation of EMT.

## Results

We analysed the genome-wide binding sites of multiple factors, including CTCF, ELF1, ETS1, FOSL2, GABPA, REST, EP300, SIN3AK20, SIX5,SP1, TAF1, TCF12, USF1, YY1, ZBTB33, FOXA1, ATF3, BCL33, and RNA polymeraseII(POL2) using ENCODE ChIP-Seq data from human A549 cells [15]. Using mRNA microarray data (GSE17708) from A549 cells treated with TGF-beta, we compared these binding site profiles with gene expression changes associated with EMT [16]. To determine which TFs play an important role during EMT in lung cancer, we calculated the potential S_g_ of each gene to be regulated by each of the 19 TFs (see Methods). For each TF we defined the gene sets M100, M200, M500 and M800 as the 100, 200, 500, or 800 genes with the highest regulatory potentials. Since the regulatory potential may be sensitive to the maximum distance between a TF binding site and the transcription start site (TSS) that would affect transcription, we considered different TF/DNA binding cut-off distances from 1 to 10 kb to TSS.

To identify genes that are differentially expressed during EMT in lung cancer, we analyzed gene expression data on human A549 cells after treatment with TGF-beta for 0, 0.5, 1, 2, 4, 8, 16, 24, and 72 h. Array quality control and data normalization were performed with a linear model for microarray data, Limma [17]. Genes with low variability across samples were removed from the analysis. The gene expression pattern displayed obvious differences after 16 h of TGF-beta induction. We therefore selected 15 microarrays and grouped them into two groups: a control group that included six samples at 0 h and 0.5 h, and a treatment group that included nine samples at 16 h, 24 h, and 72 h. The differentially expressed genes during EMT were determined with *p* values corrected with the Benjamini-Hochberg method [18] for controlling false discovery rate. We identified 2,188 upregulated genes and 1,948 downregulated genes (adjusted *p* <0.01).

### The impact of transcription factors during EMT in lung cancer

**Figure 1:**
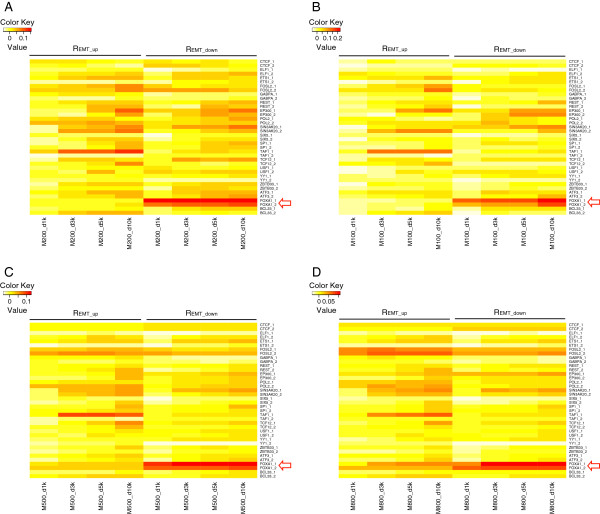
**Transcription factor (TF) impact scores of FOXA1 during epithelial-to-mesenchymal transition (EMT) in A549 lung cancer cells. (A)** The top 200 regulated genes (M200). **(B)** The top 100 regulated genes M1000. **(C)** The top 500 regulated genes (M500). **(D)** The top 800 regulated genes (M8000).

We compared the genes that had high TF regulatory potential scores with genes that were differentially expressed in EMT. Specifically, we calculated impact scores, R_EMT_up_ and R_EMT_down_, for each TF, to summarize the fraction of genes with a high regulatory potential that were, respectively, up- or down-expressed during EMT (see Methods). R_EMT_down_ for FOXA1 was significantly greater than that for other TFs in biologically replicated ChIP-Seq data for gene sets M200. Scores were statistically higher (*P* < 0.001) for FOXA1, even when the regulatory distance was varied from 1 to 10 kb (Figure 1A). We saw similar results for gene sets M100 (Figure 1B), M500 (Figure 1C), and M800 (Figure 1D), suggesting that FOXA1 is the most prominent considered TF involved in EMT. FOXA1 downregulates target genes involved in EMT at loci closer to the TSS than other TFs, since R_EMT_down_ was consistently high regardless of the regulatory distance. Conversely, R_EMT_up_ and R_EMT_down_ of other TFs (e.g., TAF1 and SIN3AK20) gradually increased as the regulatory distance to the TSS increased (Figure 1). The closer proximity of FOXA1 to the TSS further strengthens the likelihood of true transcriptional regulation, rather than non-regulatory TF/DNA association.

### Identification and functional analysis of TF-regulated targets

**Figure 2:**
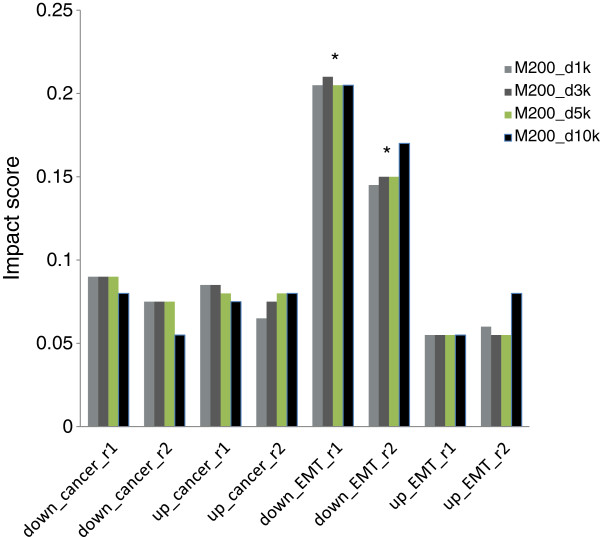
**Impact scores of FOXA1 involvement in epithelial-to-mesenchymal transition (EMT) in A549 lung cancer cells.** Impact scores for R_(ca_down)_, R_(ca_up),_ R_(EMT_down)_ and R_(EMT_up)_ in two biological replicates of ChIP-Seq data calculated for regulatory distances of 1, 3, 5, or 10 kb from the transcription start site. *p < 0.001.

To determine whether FOXA1 also contributes to lung adenocarcinoma tumorigenesis by directly regulating its transcriptional targets, we collected 1,262 upregulated and 1,262 downregulated genes in lung adenocarcinoma from Oncomine [19] gene expression signatures with concept names of ‘Lung Adenocarcinoma vs. Normal - Top 10% Over-expressed (Su Lung)’ and ‘Lung Adenocarcinoma vs. Normal - Top 10% Under-expressed (Su Lung)’. Impact scores were calculated for each TF in lung cancer tumorigenesis as R_ca_up_ and R_ca_down_. For FOXA1, R_EMT_down_ scores for different regulatory distances were significantly high, *p* < 0.001) but R_ca_up,_ R_ca_down_ and R_EMT_up_ scores were not significantly high (Figure 2). Our analysis indicates that FOXA1 is involved in EMT, but may not significantly contribute to lung adenocarcinoma tumorigenesis through direct regulation of target genes. We therefore focused on the regulatory mechanism of FOXA1 during EMT.

As FOXA1 was implicated during EMT in lung cancer, we examined which functions FOXA1 was likely to regulate. Both vertebrate liver and lung derive from the early embryonic endoderm layer and thus have similar EMT programs. Therefore, we used the consensus binding sites generated from FOXA1 ChIP-Seq data in A549 cells and hepatic HepG2 cells to narrow down a set of EMT-related and potentially FOXA1-regulated genes. MACS [20] estimates the false discovery rate of each binding site by *q* value [21]. Using a strict threshold of *q* < 10^-10^, we identified 22,554 overlapping binding sites. Looking for motifs in the top 5,000 overlapping binding sites using seqPos [22], we found that all top 10 motifs (Additional file 1: Table S1) belong to the Forkhead family, with FOXA1 motif being the most enriched (zscore = −101.69). The motif analysis suggests that FOXA1 might regulate EMT-related genes directly. We then applied the regulatory potential (S_g_) to sort FOXA1-regulated genes and selected top 200 genes (Additional file 2: Table S2) with a regulatory potential greater than 1.55 as the most likely candidates of FOXA1-regulated genes.

We employed GREAT [23] to find functional categories enriched by FOXA1-regulated genes (adjusted *p* <0.05). We then used the hypergeometric distribution to select the functions also enriched by upregulated or downregulated genes during EMT (adjusted *p* <0.05). FOXA1-regulated genes were involved in a variety of biological processes and pathways. Downregulated, but not upregulated, genes were enriched in 18 GO Biological Processes, 1 GO Cellular Component, and 41 Pathway Commons (Table 1), suggesting that FOXA1 may be repressing EMT. The EMT-related functions enriched by downregulated genes included cell communication, nectin adhesion pathways, focal adhesion kinase signaling, fibrinogen complex signaling, and FOXA1 TF networks (Table 2). The information of all enriched functions, enrichment scores, and the enriched genes was also shown (Additional file 3: Table S3).

**Table 1:** The number of functions enriched by FOXA1-regulated genes during EMT

**Functional category**	**Regulated genes**	**Upregulated genes**	**Downregulated genes**
GO Biological process	117	0	18
GO Cellular component	1	0	1
GO Molecular function	1	0	0
MSigDB pathway	8	0	0
Pathway commons	73	0	41

Functions with *p* < 0.05 are included.

**Table 2:** EMT-related functions

**Ontology**	**Description**	**Downregulated genes**	***p *** **value**
Pathway Commons	Nectin adhesion pathway	*CP(↓),DUSP1,DUSP6,EFNA1,FGA(↓),FGB(↓),FOS(↓),GDF15(↓),HES1,IKBKG,NEDD4L(↓),ODC1,PIK3CA,* *SERPINE1,SMAD3,TGIF1,VTN*	0.0207
Pathway Commons	Signaling events mediated by focal adhesion kinase	*CP(↓),DUSP1,DUSP6,EFNA1,FGA(↓),FGB(↓),FOS(↓),GDF15(↓),HES1,IKBKG,NEDD4L(↓),ODC1,PIK3CA,* *SERPINE1,SMAD3,TGIF1,VTN*	0.0207
Pathway Commons	FOXA1 transcription factor network	*FOS(↓),FOXA2(↓),NR2F2(↓),TFF1,VTN*	0.0319
GO Cellular Component	Fibrinogen complex	*FGA(↓),FGB(↓),FGL1(↓)*	0.0207
GO Biological Process	Cell communication	*ANG(↓),DHCR24(↓),DUSP1,DUSP6,ECT2(↓),EFNA1,ELK3,F2RL1,FGA(↓),FGB(↓),FGL1(↓),FOS(↓),* *FOXA2(↓),FRAT1(↓),FRAT2,GDF15(↓),GLRX2,GPR126,GPRC5A(↓),GUCA1B,HES1,HNF1A,HNF1B,* *IKBKG,KLF9,MCU(↓),NAB2,NFE2L2(↓),NR1D1,NR2F2(↓),PDK4(↓),PIK3CA,PLA2G1B,PLCD3,PPAP2B,* *RAPGEF3,RARB,SARM1,SMAD3,UBB,UBC,*	0.0006

**Figure 3:**
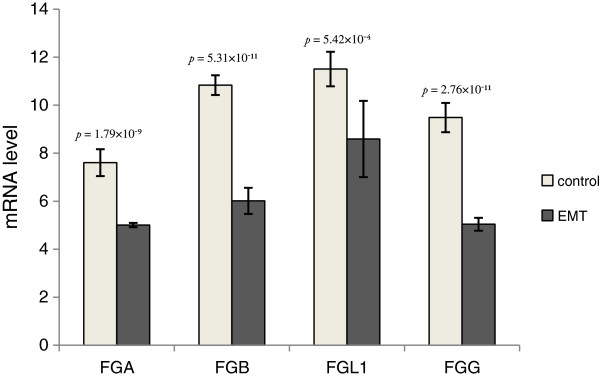
**Microarray data depicts mRNA expression levels.***FGA*, *FGB*, *FGG*, and *FGL1* mRNA expression levels in controls and epithelial-to-mesenchymal (EMT) groups.

**Figure 4:**
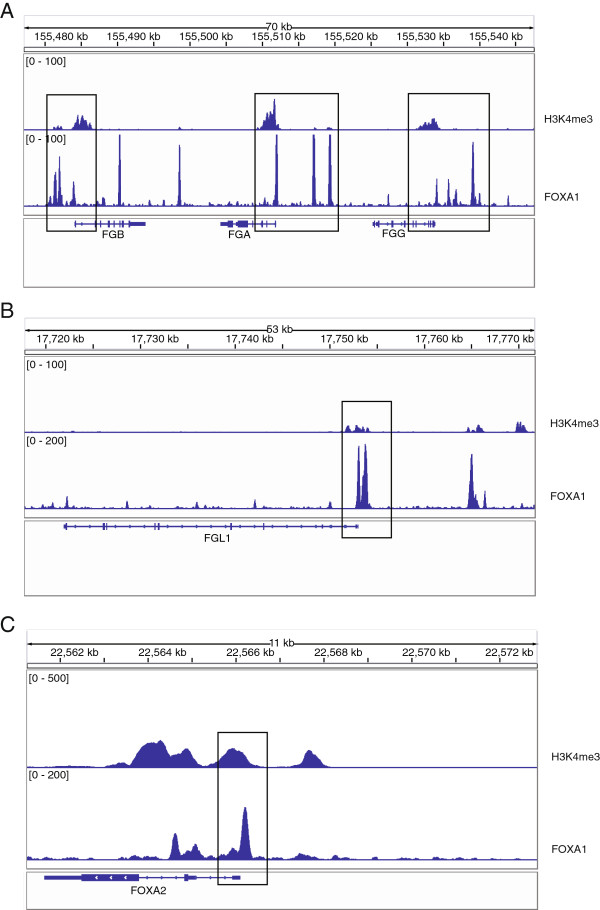
**FOXA1 binding sites in the TSS of *FGA*, *FGB*, *FGG*, *FGL1 *and *FOXA2*.** Binding sites in **A)***FGA*, *FGB*, *FGG*, **B)***FGL1* and **C)***FOXA2* were identified in A549 cells by analysis of ChIP-Seq data. The boxed sites indicate the TSS of genes.

**Figure 5:**
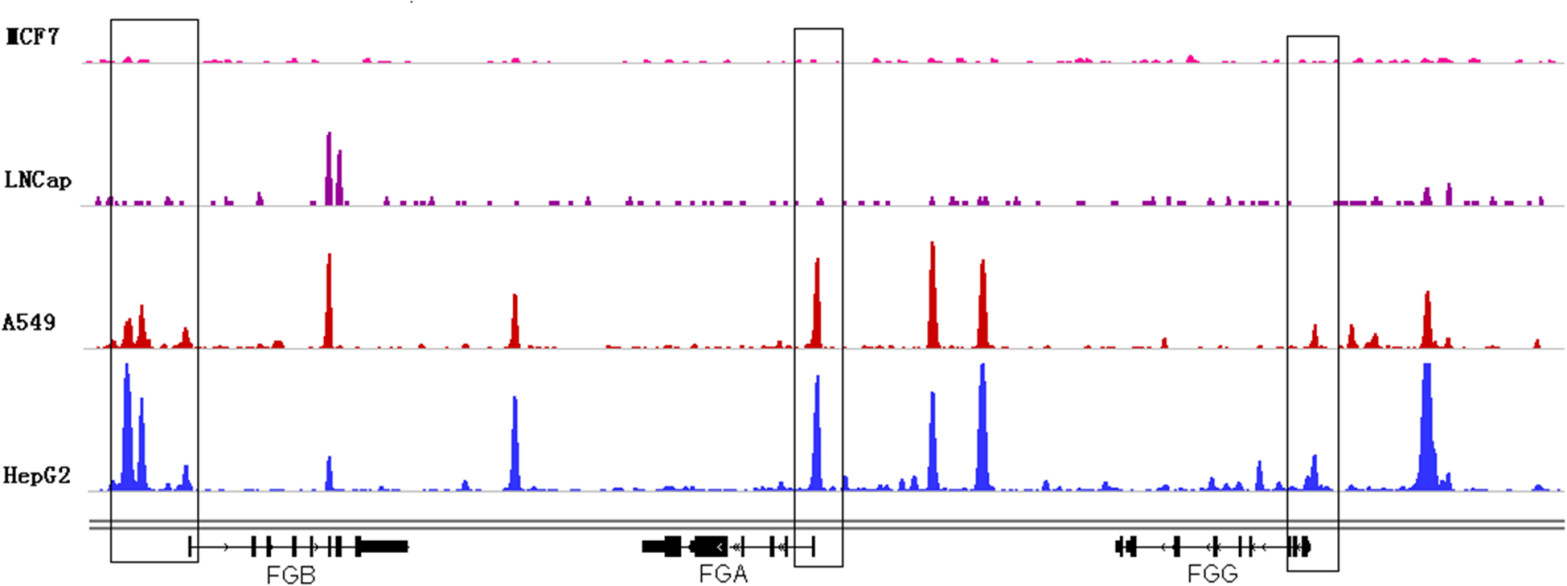
**FOXA1 binding sites in the upstream regions of *FGA*, *FGB *and *FGG.*** ChIP-Seq identified binding sites in four different cell lines are depicted: MCF7 cells, LNCaP cells, A549 cells, and HepG2 cells. The boxed sites indicate the TSS of genes.

Four downregulated genes that enriched cell communication, nectin adhesion, and focal adhesion kinase and fibrinogen complex signaling are related to the loss of epithelial traits in metastasis initiation. *FGA, FGB* and *FGL1* were involved in multiple EMT-related functions (Table 2), and *FGG* was also identified with a less stringent *q* value threshold. *FGA/FGB/FGG* encode the alpha/beta/gamma components of fibrinogen, a blood-borne glycoprotein comprised of three pairs of non-identical polypeptide chains. Fibrinogen is cleaved by thrombin following vascular injury to form fibrin, the most abundant component of blood clots [24]. In addition, fibrinogen induces endothelial cell adhesion and spreading via the release of endogenous matrix proteins and the recruitment of more than one integrin receptor [25]. *FGL1* also encodes a member of the fibrinogen family. *FGA, FGB, FGG* and *FGL1* were downregulated during EMT of lung cancer (Figure 3) and were mapped to the GO term ‘Fibrinogen complex’. ChIP-Seq data from A549 cells showed that FOXA1 was strongly bound to TSS of these genes (Figure 4A-B). No binding sites were found at the TSS of these genes in the human breast adenocarcinoma cell line MCF7 or in the human prostate adenocarcinoma cell line LNCaP (Figure 5), suggesting that the regulatory role of FOXA1 in breast and prostate cancer might differ from that of lung and liver cancer. Thus, *FGA, FGB, FGG* and *FGL1* could be important direct target genes of FOXA1 that are involved in EMT of lung and liver cancer specifically.

*FOXA2* was involved in the functions including FOXA1 transcription factor network and cell communication, and *FOXA2* was downregulated during EMT of lung cancer (Table 2). ChIP-Seq data showed that FOXA1 was bound to TSS of *FOXA2* (Figure 4C).

### Experimental validation of predicted FOXA1-regulated targets

**Figure 6:**
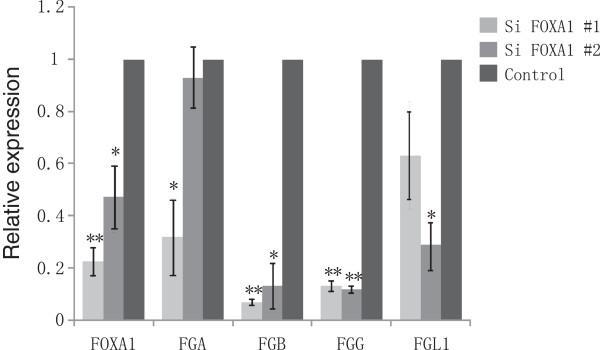
**Expression of *FGA*, *FGB*, *FGG *and *FGL1 *were downregulated by FOXA1 knockdown in A549 cells.** RT-qPCR analysis of expression of *FOXA1*, *FGA*, *FGB*, *FGG* and *FGL1* in A549 cells after transfection with FOXA1-specific siRNA (#1 and #2). * *p* < 0.05, ** *p* < 0.005 (as compared with control).

RNA interference and reverse transcription quantitative PCR (RT-qPCR) were used to check whether FOXA1 regulates its predicted target genes in A549 cells. We investigated FOXA1’s effect on *FGA*, *FGB*, *FGG* and *FGL1* genes by knocking down FOXA1 followed by RT-qPCR. Expression of *FGB* and *FGG* showed the significant decrease after FOXA1 was knocked down with two independent siRNAs targeting FOXA1, while expression of *FGA* and *FGL1* was significantly decreased after transfection with one of the siRNAs (Figure 6).

## Discussion

EMT is a physiological process originally described in embryonic development [26]. FOXA family proteins are critical for epithelial differentiation in many endoderm-derived organs, including pancreas [27], lung [28] and liver [29]. Crawford *et al.* identified the role of FOXA1 during EMT in pancreatic ductal adenocarcinoma [30]; they found that FOXA1 and FOXA2 are important antagonists of EMT through positive regulation of E-cadherin and maintenance of the epithelial phenotype. Song *et al.* also reported that FOXA2 suppresses tumor metastasis by inhibiting EMT in human lung cancer [31].

Here, we identified FOXA1 as an important TF involved in EMT during lung cancer progression. Genes regulated by FOXA1 are down-expressed and enriched in the functions including cell communication, nectin adhesion pathways, focal adhesion kinase signalling and fibrinogen complex signalling, so FOXA1 may be directly involved in metastasis initiation, namely the loss of cellular adhesion and cellular communication. Several EMT effectors, including *FGA*, *FGB*, *FGG*, and *FGL1*, were indicated as potential regulatory targets of FOXA1. Expression of *FGB* and *FGG* showed significant decrease after FOXA1 was knocked down. Therefore, our analysis combining expression and TF regulatory data suggests that FOXA1 could be the potential negative regulator of EMT and could play pivotal roles in the initial steps of lung cancer metastasis. In pancreas cancer FOXA1/2 factors are suppressed by EMT-inducing signals, such as TGFb and DNA methylation. Methylation-mediated suppression of FOXA2 leads to downregulation of EMT-related gene E-cadherin and induces EMT [30]. Stoffel et al. [32] reported that in embryonic stem cells expression of FOXA1 is reduced in the absence of FOXA2, and *FOXA1* mRNA is undetectable in FOXA2 null embryoid bodies, implying that FOXA2 is essential for FOXA1 expression. Therefore, in lung cancer FOXA1 activity may be regulated by other regulators such as FOXA2 whose activities could be directly modulated through epigenetic mechanisms. Interestingly, our study also found a strong binding site of FOXA1 at the promoter of *FOXA2* (Figure 4C), suggesting the potential regulation of FOXA1 on *FOXA2* in A549 cells. More detailed functional and mechanistic studies are required to fully unveil the significance of FOXA1 during EMT and lung cancer progression.

Our study further demonstrates how published ChIP-Seq and gene expression data could be integrated to understand the impact of TFs in a specific biological process. ChIP-Seq data of A549 cells were generated without TGF-beta stimulation, so our approach might only fish out negative regulators and might miss factors which positively regulate EMT. Despite this limitation, our approach is expected to identify potential positive factors if ChIP-Seq data from TGFb-stimulated cells are available.

## Conclusions

A systematic computational analysis based on multiple ChIP-Seq and gene expression datasets suggests that FOXA1 is a potentially important negative regulator in the EMT of lung adenocarcinoma. FOXA1 may regulate a series of critical EMT effector genes relevant to cellular adhesion and cellular communication to maintain epithelial traits downregulated in EMT. This approach can also be transplanted into other biological systems to infer regulators of transcriptional mechanisms, especially as more ChIP-Seq and expression data accumulate.

## Methods

### Datasets

Multiple ChIP-Seq and transcriptome datasets for human lung adenocarcinoma are available from ENCODE, GEO and Oncomine databases. We collected ChIP-Seq data of 18 TFs and RNA polymeraseII in human A549 cells generated by ENCODE project. ChIP-Seq data in A549 cells was generated after the treatment of 0.02% Ethanol for 1 hour. We used the GSE17708 mRNA microarray dataset from A549 cells treated with 5 ng/mL TGF-beta for 0, 0.5, 1, 2, 4, 8, 16, 24, and 72 h to induce EMT. Each ChIP-Seq experiment had two replicates; the microarray data was in triplicate. A differentially expressed gene set in lung adenocarcinoma tumorigenesis based on gene signatures from the Oncomine database was used as a control. This array (U133A) included 1,262 of the top 10% upregulated genes and 1,262 of the top 10% downregulated genes in lung adenocarcinoma compared with normal samples. In addition, FOXA1 binding site data from hepatic hepG2 cells were used.

### Regulatory gene analysis

We first used each TF’s binding sites to define their regulatory genes with a previously described algorithm [33]. The sum of the nearby binding sites weighted by the distance from each site to the TSS of a given gene was used to calculate the regulatory potential S_g_ for each gene:

$$S_{g}=\sum_{i=1}^{k}e^{-\left(0.5+4\Delta_{i}\right)},$$

Where k is the number of binding sites within the regulatory distance of gene g and Δ_i_ is the distance between site i and the TSS of gene g. For regulatory genes prediction, we respectively considered genes with at least one binding site within 1 kb, 3 kb, 5 kb and 10 kb from its TSS. By combing differentially expressed genes in lung cancer EMT from transcriptome data, we gave each TF an impact score based on the regulated genes’ involvement in EMT. We assumed that genes regulated by one TF were differentially expressed if that TF was involved in EMT. With this assumption, we first overlapped TF-regulated genes and upregulated or downregulated genes and then used the ratio of overlapping genes to total numbers of regulated genes as a measure of TF impact:

R=nent,

where R is TF impact score, n_e_ is the number of overlapping genes , and n_t_ is the number of total target genes. Impact score is large when TFs play a role in EMT.

We used the hypergeometric distribution to assess the statistical significance of R, where the basic definition of the hypergeometric distribution of a random variable X is:

$$p\left(X=x|N,M,n\right)=\frac{\left(\begin{array}{c} M\\ x \end{array}\right)\left(\begin{array}{c} N-M\\ n-x \end{array}\right)}{\left(\begin{array}{c} N\\ n \end{array}\right)},$$

where N is the number of all genes, M is the number of TF regulated genes, n is the number of upregulated or downregulated genes in EMT, and x is the number of upregulated or downregulated genes in the top M. Whether the p value of the random variable X is greater than a specific value x can be calculated by:

$$p\left(X\geq x|N,M,n\right)=1-\overset{x-1}{\underset{i=0}{\sum }}\frac{\left(\begin{array}{c} M\\ i \end{array}\right)\left(\begin{array}{c} N-M\\ n-i \end{array}\right)}{\left(\begin{array}{c} N\\ n \end{array}\right)},$$

For the TF with the highest R value, we further employed other ChIP-Seq data to winnow binding sites and determine the most likely regulatory genes.

### Functional analysis

Function annotation frames GO, MSigDB pathway, and Pathway Commons were used to select the significant functions enriched by TF regulatory genes with cis-regulatory regions function analysis tool GREAT. A hypergeometric distribution was used for each of the enriched functions to assess whether the TF-enriched functions were also enriched by differentially expressed genes during EMT. If they were, these functions were assumed to be EMT-related. The *p* value was calculated as:

$$p\left(X\geq x|N,M,n\right)=1-\sum_{i=0}^{x‒1}\frac{\left(\begin{array}{c} M\\ i \end{array}\right)\left(\begin{array}{c} N‒M\\ n‒i \end{array}\right)}{\left(\begin{array}{c} N\\ n \end{array}\right)},$$

where N is the number of all genes, M is the number of TF regulatory genes mapped in one functional category, n is the number of upregulated or downregulated genes during EMT, and x is the number of upregulated or downregulated genes in one functional category.

### RNA interference and Quantitative real-time PCR

siRNA targeting FOXA1 was purchased from Ribobio. The sequence of siRNA #1 is: 5’- CCGGUCAGCAACAUGAACU dTdT -3’ and 5’-AGUUCAUGUUGCUGACCGGdTdT-3’; the sequence of siRNA #2 is 5’-ACGAACAGGCACUGCAAUA dTdT -3’ and 5’UAUUGCAGUGCCUGUUCGUdTdT-3’. Gene specific and scramble (control) siRNA were transfected into A549 cells using lipofectamine 2000 according to manufacturer’s instruction (Invitrogen).

Total RNA of the cells was extracted using RNAprep Pure Cell/Bacteria kit (Tiangen). 5ug of total RNA was reverse transcribed with RevertAid strand cDNA synthesis kit according to the manufacturer’s instruction (Thermo Scientific). Real time PCR was run on Applied Biosystem 7500 with GREAT Real Time SYBR PCR kit (Biovisual Lab). Expression level of each gene was normalized to GAPDH expression, and further normalized to control group. All PCR experiments were done in triplicates within each experiment. The primer sequences for FOXA1 were 5’ –GCAATACTCGCCTTACGGCT-3’ and 5’- TACACACCTTGGTAGTACGCC-3’, for GAPDH were 5’-GAGTCAACGGATTTGGTCGT-3’ and 5’-TTGATTTTGGAGGGATCTCG-3’, for FGA were 5’-AGACATCAATCTGCCTGCAA-3’ and 5’-TCAATCAACCCTTTCATCCTG-3’, for FGB were 5’-CCCAGACCTCCTCTTCTTCC-3’ and 5’-TGGTGCTTTTCCAGTTCTGA-3’, for FGG were 5’-GGAAGACTGGAATGGCAGAA-3’ and 5’-ATCATCGCCAAAATCAAAGC-3’, for FGL1 were 5’-TCCTGGGAAGCAGAGTGTCT-3’ and AAGAGCGGTGGTAACAAGGA-3’.

## Abbreviations

ChIP-Seq: Chromatin immunoprecipitation-sequencing; EMT: Epithelial-to-mesenchymal transition; TF: Transcription factor.

## Competing interests

The authors declare that they have no competing interests.

## Authors’ contributions

HW and XSL conceived the study, designed and performed the statistical analyses, and drafted the manuscript. CM and TF discussed, revised and approved the manuscript. FZ and GW designed and performed the experiment of RNA interference and Quantitative real-time PCR, and drafted the experiment section of the manuscript. All authors read and approved the final manuscript.

## Supplementary Material

Additional file 1: Table S1 The motifs in the top 5,000 binding sites analysed with motif algorithm ‘seqPos’. The top ten motifs are showed in the table. Click here for file

Additional file 2: Table S2 FOXA1 regulatory potential score as well as gene expression in TGF-beta stimulated cells of 0 h and 0.5 h time points (control) against 16-72 h (EMT) for all genes. ‘NA’ means there is no expression data for that gene. Top 200 genes are selected as the predicted FOXA1 target genes. Click here for file

Additional file 3: Table S3 The information of all enriched functions, enrichment scores, and the enriched genes. Click here for file
